# A Case of Two Brothers With Dysphagia Due to Eosinophilic Esophagitis

**DOI:** 10.7759/cureus.33181

**Published:** 2022-12-31

**Authors:** Tomas Escobar Gil, Alejandro Echavarria Cross, Juan P Escobar Gil

**Affiliations:** 1 Medicine, Universidad CES, Medellín, COL

**Keywords:** atopy, biopsy, endoscopy, genetics, gastroenterology, eosinophilic esophagitis

## Abstract

Eosinophilic esophagitis (EoE) is a disease that is still not fully understood. Its pathogenesis, while increasingly clarified, still remains highly complex, which means that no curative treatment has been defined for this clinical entity. It is clear that it is a disease of multifactorial etiology, in which both genetics and environmental factors, especially those related to childhood, have considerable weight, and there is an important allergenic factor as well. We present the case of two brothers with EoE. Two male patients aged 20 and 22 years, white, with a personal history of atopy, allergic rhinitis, and dermatitis, consulted the gastroenterologist for dysphagia. Endoscopy and esophageal biopsy showed elements compatible with EoE in both of them. Treatment was conducted with proton pump inhibitor (PPI) monotherapy in one of the brothers, and PPI with oral steroid in the other, both of which led to good results in terms of symptoms. In the first case, histologic evidence of the disease persisted despite the symptomatic resolution; the second did not pursue a follow-up. The biggest questions pertaining to the treatment of this condition are as follows: Is suppression of gastric acidity enough? Should we use steroids? How about a combination of both? Should we adopt new therapies? New studies involving randomized trials should be conducted to address these questions in order to treat each patient individually with an effective and practical approach that is also supported by the literature.

## Introduction

Eosinophilic esophagitis (EoE) is a complex genetic disorder characterized by eosinophilic inflammation within the esophagus [1]. It is defined by symptoms of esophageal dysfunction such as emesis, dysphagia, or feeding difficulties, in patients with an esophageal biopsy demonstrating at least 15 eosinophils/high power field, in the absence of other conditions associated with esophageal eosinophilia such as gastroesophageal reflux disease or achalasia [2].

It is a relatively new disease, first described in the 1990s, and its incidence and prevalence have been on the rise in recent years [3,4]. In the United States, it is estimated that there are about 150,000 people with EoE [2]. In 2019, a multicenter study of pediatric patients in hospitals in Latin America found a prevalence rate in that continent of 3.69 cases × 1,000 [5]. Another study in Colombia in 2019 revealed similar findings [6]. It is estimated that 56 new cases per 100,000 people are diagnosed each year [3].

EoE is most frequently seen in Caucasian males and individuals of European ancestry, highlighting a genetic etiology of the disease. EoE has often been observed to occur in multiple family members, particularly siblings, in a non-Mendelian pattern, indicating that the hereditary component of EoE is likely complex and multifactorial in nature [1,7].

Although EoE has not been shown to increase the risk of esophageal cancer or to increase mortality, this disease significantly affects the quality of life, especially in those patients for whom it is symptomatic [3]. When this occurs, it manifests with symptoms suggestive of gastroesophageal reflux such as heartburn, or in more severe cases, with dysphagia or food impaction [8]. There are even studies that indicate that patients with EoE are more likely to develop comorbid mental pathologies [9].

The treatments commonly described for EoE are oral steroids, proton pump inhibitors (PPIs), and dietary changes, among others [2,3,7,8,10-13], but the most frequently employed are the first two.

We present the case of two male siblings with a personal history of allergic rhinitis, atopy, and specific pharmacological allergies, who developed symptoms of dysphagia in the third decade of life. Upon consultation with the gastroenterologist, both underwent digestive endoscopies, which established the diagnosis of EoE in one of the brothers, and EoE comorbid with Helicobacter pylori infection in the other.

## Case presentation

Case 1

The patient was a 20-year-old male, who two years earlier had consulted for intermittent symptoms of heartburn and dysphagia; at that time, an endoscopy had been ordered and reported as normal. Subsequent to this consultation, the symptoms had not responded to non-pharmacological measures, and hence the patient decided to re-consult gastroenterology two years later. The patient denied symptoms of precordial pain, weight loss, or fever.

His relevant history included dermatitis, allergic rhinitis of several years of evolution, and aspirin allergy that had manifested with angioedema and urticaria during childhood. The family history was generally negative at the time of initial evaluation, and both of his parents had undergone previous endoscopies with normal results; the patient reported having two brothers and two sisters, in good health, and both of his parents were alive, and also asymptomatic. The family was of southern European descent, specifically from the Iberian peninsula, and the patient had been born and raised in Colombia. Physical examination was completely normal. Routine laboratory tests, which included complete blood count, serum electrolytes, liver tests, and thyroid function tests, were also normal.

**Figure 1:**
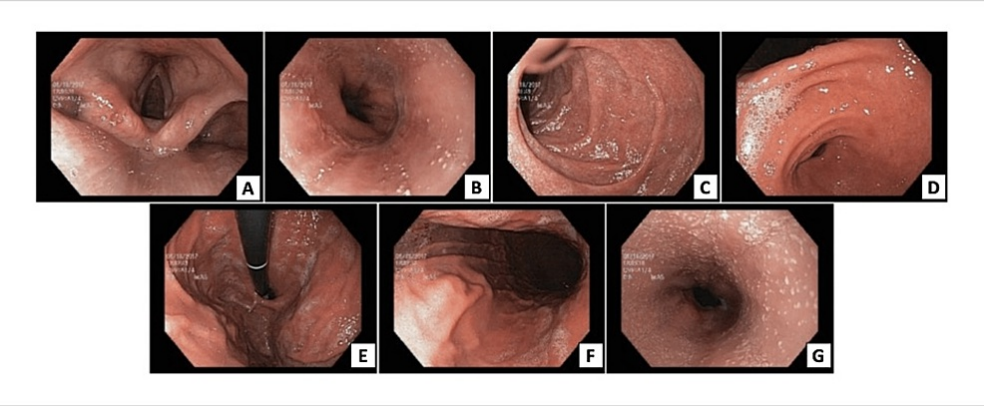
First endoscopy of patient 1 The images show focal exudates (1A), multiple whitish punctate lesions throughout the esophagus (1B, G), mucosal involvement with signs suggestive of loss of vascularization (1A-G), and esophageal grooves and rings (1C-D)

A control upper gastrointestinal endoscopy was also ordered (Figure 1); esophagoscopy showed multiple whitish punctate lesions throughout the esophagus, mucosal involvement with signs suggestive of loss of vascularization, esophageal grooves, and esophageal rings. Gastroscopy showed involvement of the antral mucosa with areas of focal erythema with no other findings. Duodenoscopy was normal.

**Figure 2:**
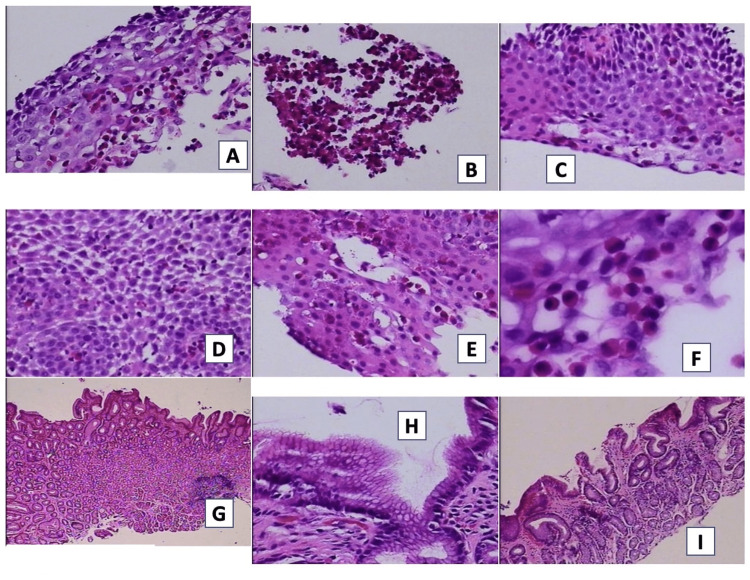
First biopsy of patient 1 The images show evidence of squamous epithelium with spongiosis, reactive changes, and the presence of intraepithelial eosinophils with microabscess formation, counting up to 43 eosinophils per high magnification field (2A-F). Chronic gastritis without activity is observed (2G-I)

Three esophageal biopsies and three gastric antral biopsies were taken. The esophageal biopsy showed squamous epithelium with spongiosis, reactive changes, and the presence of intraepithelial eosinophils with microabscess formation, counting up to 43 eosinophils per high magnification field. The study was negative for dysplasia, intestinal metaplasia, or Helicobacter pylori. Stomach biopsies revealed mild chronic gastritis without activity, with no other relevant findings. Based on the above findings, a diagnosis of EoE was determined (Figure 2).

**Figure 3:**
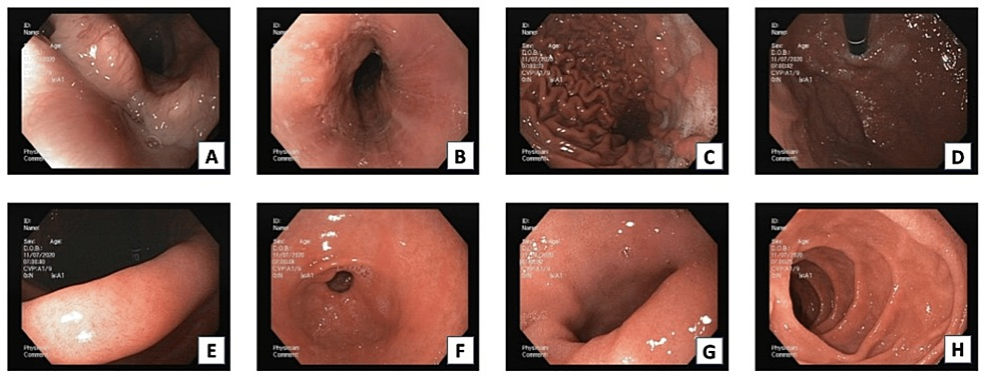
Second endoscopy of patient 1 Focal exudates (3A-C), the persistence of rings and grooves (3B, D, H), edema (3E-G), and focal erythema (3D) are evident

**Figure 4:**
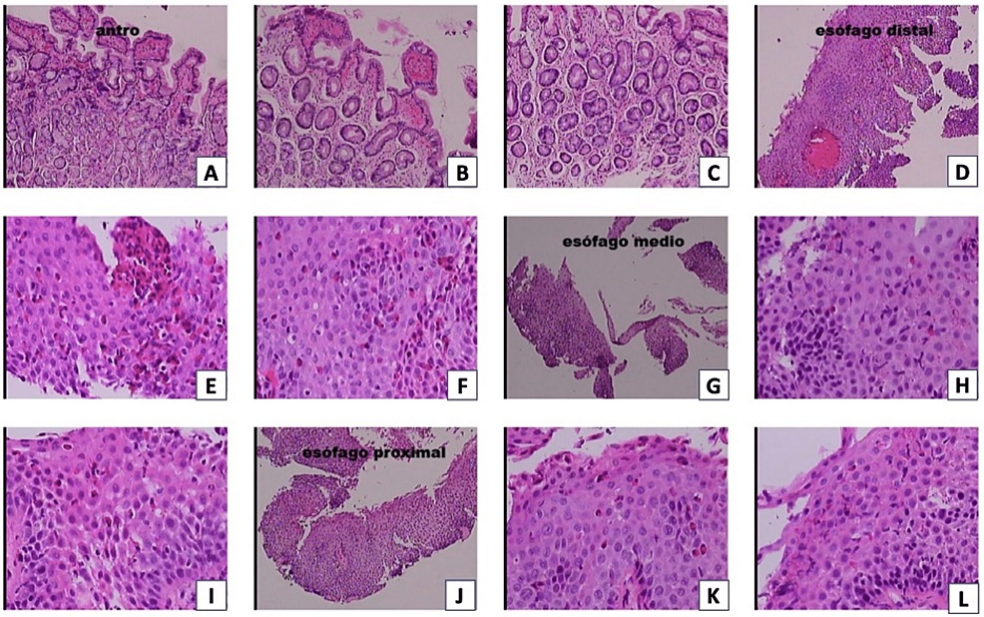
Second biopsy of patient 1 Severe inflammation (4D-L), eosinophilic infiltrates (4D-L), high magnification field count of more than 60 eosinophils in distal esophagus (4D-F), 23 eosinophils in the middle esophagus (4G-I), and 30 in the proximal esophagus (4J-L), and edema and congestion are also observed. Lymphocytes are also found in the middle esophagus (4G-I)

The patient started therapy with omeprazole twice a day with marked symptomatic improvement and returned for a follow-up appointment after two years, at which a control endoscopy was scheduled. The endoscopy (Figure 3) showed whitish plaques, the persistence of the grooves and rings from the previous endoscopy, and foci of erythema. The biopsy (Figure 4) revealed severe inflammation, eosinophilic infiltrates, a high magnification field count of more than 60 eosinophils in the distal esophagus, 23 eosinophils in the middle esophagus, and 30 in the proximal esophagus; edema and congestion were also observed. Lymphocytes were also found in the middle esophagus.

Since the patient was asymptomatic following the established treatment, it was decided to continue only with PPI therapy, and resume the usual follow-up.

Case 2

The patient was a 22-year-old male who consulted for dysphagia, associated with a sensation of esophageal strictures with food, retrosternal pain predominantly in the morning, pyrosis, and odynophagia. The patient denied weight loss or fever. As for his history, egg allergy was found to be relevant, which manifested with a reaction to an influenza vaccine in childhood, in addition to a history of dermatitis and allergic rhinitis of several years of evolution. A family history of EoE was reported in his brother (Case 1), but both parents had undergone previous endoscopies with normal results. Physical examination was completely normal. Routine laboratory test results, which included hemogram, ionogram, liver tests, and thyroid function tests, were normal. The only abnormal finding was a positive breath test for Helicobacter pylori.

**Figure 5:**
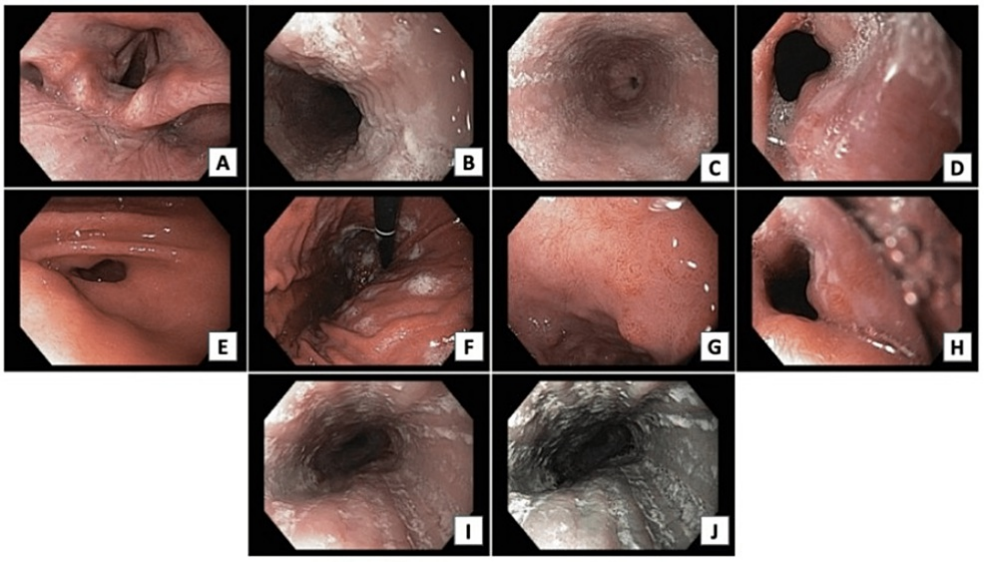
Endoscopy of patient 2 The images show linear lesions smaller than 5 mm (Grade A) (5F), whitish-yellowish exudates throughout the organ that were easily detached (5B, C, D, F, H-J), and focal areas of inflammation (5A, E, G, H)

A digestive endoscopy was also ordered in which the following was found (Figure 5): esophagoscopy showing linear lesions smaller than 5 mm (Grade A), and whitish-yellowish exudates throughout the extension of the organ that were easily dislodged. No esophageal rings or grooves, dysplasia, intestinal metaplasia, or other findings were described.

On gastroscopy, focal erythematous areas were described in the antral region of the stomach, and on duodenoscopy, foci of congestion were described. The diagnostic impression was erosive gastritis Grade A based on the Los Angeles classification, esophageal candidiasis versus EoE, antral erythematous gastritis without activity, and mild focal congestive bulbo-duodenitis. Six biopsies (three of the antrum and three of the distal esophagus) were taken and sent to pathology, and empirical therapy was started with fluconazole (for suspected candidiasis), and clarithromycin, omeprazole, and amoxicillin (due to the positive breath test). An HIV test was also requested and was negative.

**Figure 6:**
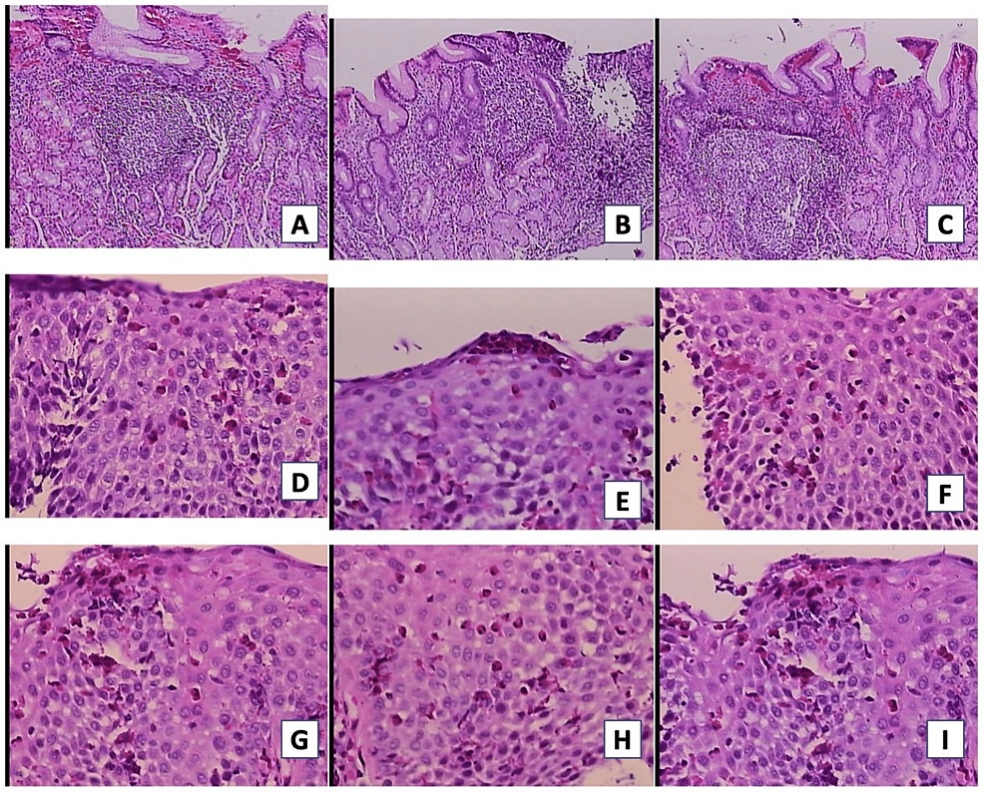
Biopsy of patient 2 The images show severe inflammation of the esophagus, eosinophilic infiltrates, high magnification field count of more than 20 eosinophils per high power field, scarce Candida pseudohyphae (6D-I); and in the stomach, moderate inflammation, with infiltrates of macrophages, lymphocytes, and plasmocytes, and Helicobacter pylori in moderate amount (6A-C)

The results of the biopsies (Figure 6) were as follows: in the distal esophagus, severe inflammation, eosinophilic infiltrates, high magnification field count of more than 20 eosinophils per high power field, and scarce Candida pseudohyphae; and in the stomach, moderate inflammation, with infiltrates of macrophages, lymphocytes, and plasmacytes, and Helicobacter pylori in moderate quantity. The diagnoses of EoE, chronic follicular gastritis, and helicobacter Pylori infection were made.

The patient continued to use omeprazole twice a day. He reported complete resolution of symptoms at the end of treatment. He clarified that his symptoms had responded to PPIs after the event, and he has not had any follow-up endoscopies.

## Discussion

In 2005, Patel and Falchuk published the first case report of EoE in siblings [11]. More information is now available about the implications of this disease, which was first described in detail in 1993 [11], and the role of genetics in its pathogenesis has become clearer [13-17]. After that first study, other reports of EoE in siblings have been published [10,18,19].

The high prevalence of EoE among individuals of European descent and men observed in multiple epidemiological studies implies a genetic aspect in the etiology of the disease [1]. It has been reported that up to 6.8% of patients with EoE have a family history of the disease [10]. Moreover, it appears that early life exposures modify the risk of EoE, with maternal fever, prenatal and postnatal antibiotics, PPI therapy, and neonatal ICU admission associated with increased risk of EoE and pet exposure in the first year of life associated with decreased risk [19].

How then do genes influence pathogenesis? There is no doubt that EoE is a disease of multifactorial etiology, but it is also known that there is a clear genetically driven allergic component, as not only eosinophilic infiltration but also the presence of TH2 cells, interleukin-5 (IL-5), IL-13, and eotaxin-3 have been identified in biopsies, implying that, in at least a segment of patients, the etiology of inflammation is allergic [1,7]. A subgroup of patients, despite having eosinophilic infiltration and esophageal inflammation, have shown no known allergens and no history of allergic disease, again suggesting that multiple factors (both environmental and genetic) influence susceptibility to disease and its development [1]. At least two genes on chromosome 5 have been described that could be involved in the pathogenesis of the disease (TSLP and WD36) [1,16], as well as one gene on chromosome 2 (CAPN14) [16].

The diagnosis of this entity continues to rely on endoscopy with six esophageal biopsies [20], with follow-up after diagnosis to assess histologic response [2]. Both cases in this report presented pathognomonic macroscopic findings of the disease.

Tools have been proposed to measure the severity of the disease, such as the Dellon et al. score [21], which combines clinical, symptomatic, endoscopic, and laboratory findings; however, its usefulness continues to be debated and studied. Similarly, Shoda et al. [22] proposed disease phenotypes based on endoscopic and histologic findings. The endoscopic reference score (EREFS), proposed in 2013 by Hirano et al., is used to determine the severity of five endoscopic findings: edema, rings, exudates, furrows, and strictures [23]. Each of the five components is given a number or a score. A higher total score (when all five component scores are added together) may reflect more severe or serious EoE.

For Case 1, the EREFS was calculated as follows: edema was given a score of 2 (severe) as there was a loss of vascular margins; fixed rings were present and were given a score of 2 (moderate) as they did not impair the passage of the endoscope; exudates were given a score of 2 (severe), as they compromised more than 10% of the esophagus area; furrows were present and were given a score of 1 (mild), as they consisted only of vertical lines present without visible depth; and since there were no strictures at all, the stricture criteria was given a score of 0. The total score was 7. These findings reflect a case of severe EoE. The scoring is especially helpful in cases as severe as this because it helps objectify endoscopic findings and highlights the importance of getting adequate treatment. Follow-up endoscopies would also be helpful in such cases to monitor progression or improvement.

As for Case 2, the EREFS was calculated as follows: edema was given a score of 1 (mild) as vascular margins were easily appreciated; fixed rings were present and were given a score of 1 (mild) as they consisted of subtle circumferential ridges; exudates were given a score of 1 (mild), as they compromised less than 10% of the esophagus area; furrows were present and were given a score of 1 (mild), as they consisted only of vertical lines present without visible depth; and since there were no strictures at all, the stricture criteria was given a grade of 0. The total score was 4. These findings reflect a case of mild EoE.

Analyzing the treatment used in these two cases, PPIs and oral steroids continue to be the gold standard for EoE. In the patients in this study, symptom control was achieved mainly with PPIs as monotherapy, and although it resolved the symptoms completely, there was an increase in eosinophils in the control biopsy in Case 1, compared to the initial diagnosis. The patient in Case 2 did not follow up at all. Stepping up treatment until histologic remission is currently established as the best course of action. This was not done in this instance, and no additional follow-up endoscopies were performed. As noted with the EREFS of Case 1, it is a case that was determined to be severe, which makes the follow-up of this patient and the escalation of treatment even more imperative.

On the other hand, recently, a new molecule, dupilumab, was approved by the Food and Drug Administration (FDA) in May of 2022, under the name Duxipent®. It is the first treatment for EoE approved in the US. It is a monoclonal antibody targeting IL-4 and IL-13, which binds to the alpha subunit of the IL-4 receptor (IL-4Rα), making it a receptor antagonist [24]. It is given subcutaneously on a weekly basis, and it has been proven to reduce eosinophils in the esophagus and improve symptoms in patients with EoE. The availability of this relatively new medication in developing countries such as Colombia, where the patients in this report reside, is still uncertain. However, it would be a good adjunct to treatment in such cases.

## Conclusions

EoE is a disease that is still not fully understood. Its pathogenesis, although increasingly clarified, is highly complex, and hence no curative treatment has been defined for this clinical entity. It is clear that it is a disease of multifactorial etiology, in which both genetics and environmental factors, especially those related to childhood, have great weight, and in which there is an important allergenic factor. The biggest questions still remain unresolved: What role will dupilumab play in the treatment of EoE? Will it be readily available in developing countries? In EoE, each patient has unique and individual characteristics, and treatment response varies widely. The above-mentioned factors underline the need of adopting a step-by-step therapy plan that ensures both symptomatic relief and histologic disease remission.
